# Rare Earth Elements (REEs) Adsorption and Detoxification Mechanisms in Cell Wall Polysaccharides of *Phytolacca americana* L.

**DOI:** 10.3390/plants12101981

**Published:** 2023-05-15

**Authors:** Yingying Guo, Keyi Chen, Shihan Lei, Yuan Gao, Shengpeng Yan, Ming Yuan

**Affiliations:** Key Laboratory of Green Chemical Engineering Process of Ministry of Education, School of Environmental Ecology and Biological Engineering, Wuhan Institute of Technology, Wuhan 430205, China

**Keywords:** rare earth elements, cell wall, polysaccharides, functional groups, *Phytolacca americana*

## Abstract

The cell wall (CW) is critical for the accumulation of heavy metals in metal-tolerant plants. Polysaccharides, the main component of the CW, contribute significantly to the immobilization of heavy metals. However, the mechanisms of rare earth elements (REEs) adsorption and detoxification by polysaccharides in the cell walls of *Phytolacca americana* L. (*P. americana*) remain unclear. In this work, we explored the binding sites of REEs and the modifications to polysaccharides in the cell walls of roots and leaves in *P. americana*, in order to elucidate the adsorption and fixation mechanism of REEs by the cell wall. Our findings indicated that up to 40.7% and 48.1% of cell-wall-bound REEs were present in the root and leaf pectin, respectively. The removal of pectin led to a 39.8% and 23.6% decrease in the maximum adsorption of REEs in the CW, suggesting that pectin was the main binding site for REEs in the cell walls of *P. americana*. Hydroxyl (-OH) and carboxyl (-COOH) groups in the cell wall interacted mainly with REEs ions under stress conditions, which played a key role in REEs binding. An obvious REEs fractionation was found during the various fractions of the CW, and all fractions of the root cell wall were enriched with HREEs, whereas all fractions of the leaf cell wall were enriched with LREEs. Moreover, *P. americana* modulated cell wall composition in reaction to REEs stress. In conclusion, cell wall pectin is the main binding site of REEs, and the functional groups on the cell wall play a significant role in the binding of REEs. At the same time, plants can control the selective adsorption and fixation of REEs by adjusting the composition of cell walls. This study offers valuable insights into the mechanisms of REEs adsorption and fixation in cell walls of *P. americana*, contributing to a theoretical basis for the bioremediation of REEs pollution.

## 1. Introduction

Rare earth elements (REEs), which comprise 15 lanthanides in addition to scandium and yttrium, can be broadly categorized into two groups: light REEs (LREEs) from La to Eu, and heavy REEs (HREEs) from Gd to Lu [1]. With their remarkable properties, REEs have become indispensable components in numerous advanced technologies and renewable energy sources, such as high-powered magnets, electric vehicle batteries, and medical equipment [2,3,4]. Furthermore, several different REEs compounds have been widely used in agriculture due to their significant role in improving crop yield and quality in fertilizers [1,5]. However, the extensive exploitation of REE deposits and the use of REE-containing fertilizers have led to a rise in REE concentrations in soil environments [6,7], which potentially poses a risk to human health via the food chain [8,9]. Consequently, recovering and reutilizing REEs from contaminated soils can mitigate REE pollution and environmental toxicity.

Phytoextraction is an environmentally friendly and cost-effective method for remediating contaminated soils or low-grade mines using hyperaccumulating plants, which selectively absorb specific metals and pollutants from the above-ground biomass [10,11]. Although numerous plant species, including ferns, are known to accumulate rare earth elements (REEs) [12,13], these plants have not been fully domesticated and tend to grow slowly [14]. In contrast, *Phytolacca americana* (*P. americana*) presents an attractive option for REE hyperaccumulation due to its widespread distribution and fast growth [15,16]. Despite the potential of *P. americana* for REE hyperaccumulation, research on the uptake and accumulation processes of REEs in this plant species is scarce. It is reported that the roots of *P. americana* could uptake large amounts of REEs, as LREEs pass through Ca ion channels while HREEs pass through the same transporter proteins as Al [17]. However, the mechanisms of REEs uptake in the root of *P. americana*, especially the different components of the roots, are still not clear.

The plant cell wall serves as the main barrier against metal entry and is primarily composed of pectin, cellulose, hemicellulose, and glycoproteins [18,19,20,21]. The chemical functional groups in these components, such as -COOH and -OH, have a high affinity for metal cations, resulting in a high cation exchange capacity [22,23]. Previous research has shown that heavy metals are stored in the cell wall and that it plays a significant role in their uptake and accumulation by plants. For instance, in *Elodea canadensis*, approximately 60–65% of the total root Cd is bound to the cell wall [24]. The abundance of free carboxyl groups in pectin is widely regarded as an important binding site for metals in the cell wall of wheat, *Silene paradoxa*, and *Sedum alfredii* [25,26,27]. Nonetheless, hemicellulose has been found to adsorb more Cd than pectin in *Arabidopsis thaliana* and *Oryza sativa* L [28,29]. Both pectin and hemicellulose play critical roles in metal binding in the cell wall [30,31]. The content of cell wall polysaccharides changes during plant responses to heavy metal stress, with metal binding leading to the modification of pectin, cellulose, and hemicellulose. For example, under Al stress, total sugars and galacturonic acid content in wheat cell walls’ pectin, cellulose, and hemicellulose increased substantially [32]. Similarly, under Cd stress, the content of hemicellulose and pectin in the cell walls of *Avicennia marina*, *O. sativa*, and *Kandelia obovata* increased [27,33]. These findings revealed that cell wall polysaccharide fractions play a significant role in metal accumulation in plants. However, the knowledge regarding the modifying role of cell wall polysaccharide fractions in the accumulation of REEs in *P. americana* is currently limited.

In this study, we examined the mechanisms of adsorption and fixation of REEs by polysaccharides in the cell walls of *P. americana*. To this end, we extracted the cell wall of *P. americana* and isolated its polysaccharide fraction for experimentation. The primary aims of this study were: (1) to identify the primary binding sites of REEs in the cell wall of *P. americana*, (2) to determine the potential functional groups involved in the binding of REEs in the cell wall of *P. americana*, and (3) to investigate the response of total sugars and galacturonic acid in the cell wall of *P. americana* to REEs exposure. The outcomes of this study provide insights into the adsorption and tolerance mechanisms of REEs and offer a theoretical basis for the bioremediation of REEs pollution.

## 2. Results

### 2.1. REEs Accumulation in Various Fractions of Root and Leaf Cell Walls of P. americana

**Figure 1:**
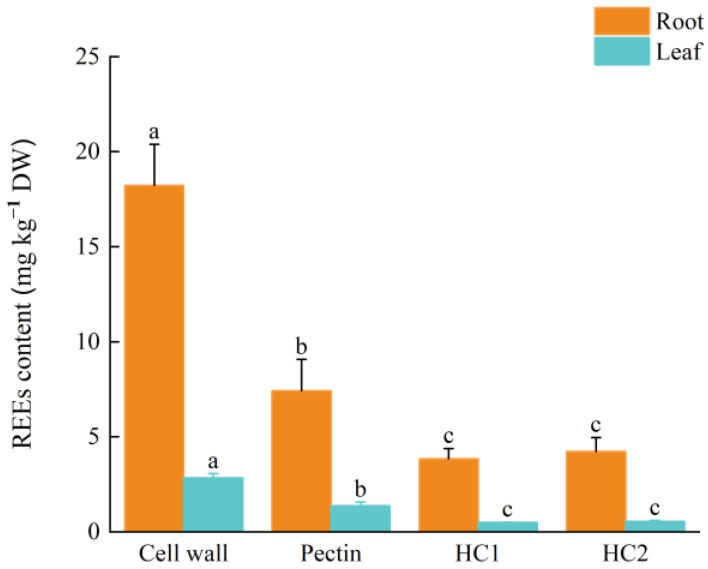
REEs content (from La to Lu) in different fractions (Pectin, Hemicellulose 1 (HC1), Hemicellulose 2 (HC2)) of root and leaf cell wall (means ± SD). Letters denote statistical significance (ANOVA, *p* < 0.05).

Based on the analysis presented in Figure 1, the most REEs were accumulated in pectin, with 41% of the total REEs in the root cell wall, followed by HC2 and HC1, the REEs accumulation of which accounted for 23% and 21% of the total REEs in the root cell wall, respectively. Similarly, in the leaf cell wall, pectin accumulated the most REEs, accounting for 48% of the total REEs. In addition, the REEs accumulation of HC2 and HC1 accounted for 20% and 17% of the total REEs in the leaf cell wall, respectively. Furthermore, the analysis revealed that the root cell walls have a significantly higher binding capacity for REEs compared to leaf cell walls. Specifically, the accumulation of REEs in root cell wall pectin, HC1, and HC2 were 5, 8, and 8 times higher than those in leaf cell walls, respectively. These findings suggest that pectin is the primary binding site of REEs in both the root and leaf cell walls of *P. americana*.

### 2.2. REEs Fractionation in Different Fractions of the Cell Wall of P. americana

**Figure 2:**
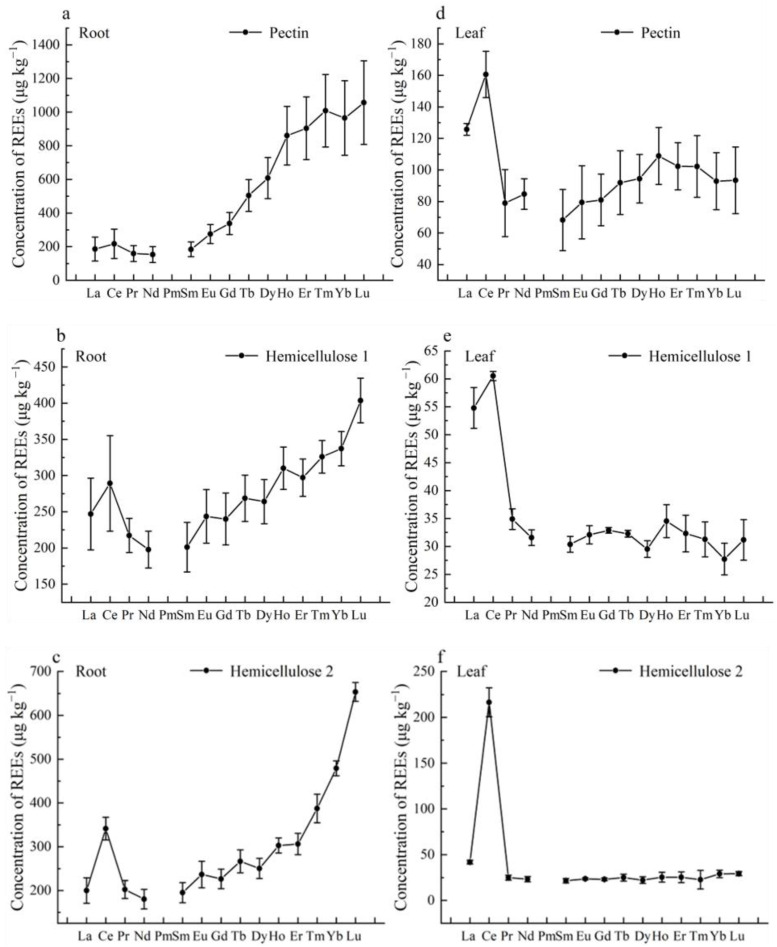
REEs concentration in different polysaccharide fractions of the cell walls of root (**a**), Pectin; (**b**), Hemicellulose 1; (**c**), Hemicellulose 2 and leaf (**d**), Pectin; (**e**), Hemicellulose 1; (**f**), Hemicellulose 2 in *Phytolacca americana*.

**Figure 3:**
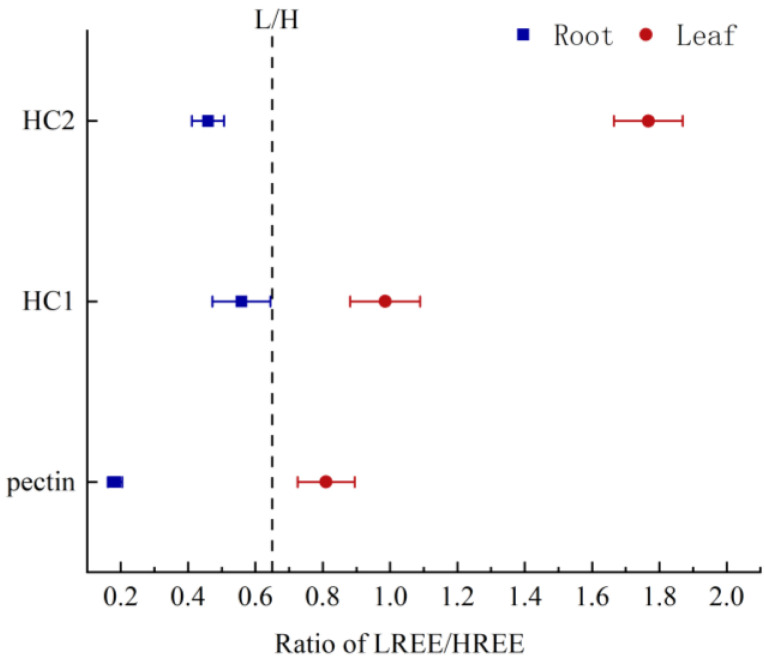
The ∑LREE/∑HREE values in different components (Pectin, Hemicellulose 1 (HC1), Hemicellulose 2 (HC2)) of root and leaf cell wall of *Phytolacca americana* (means ± SD). The black dash line is the standard value of ∑LREE/∑HREE (mass ratio of LREEs to HREEs, each element under equal molar concentration, lower than standard value means HREEs enrichment and vice versa).

Figure 2 shows that all fractions of the root cell wall were enriched with HREEs, whereas all fractions of the leaf cell wall were enriched with LREEs. Notably, pectin in the root cell wall exhibited the greatest enrichment of HREEs, while HC2 in the leaf cell wall exhibited the highest enrichment of LREEs. The ∑LREE/∑HREE values in different components of the root cell wall decreased in the order of HC1 > HC2 > pectin, with values of 0.56, 0.46, and 0.18, respectively. Similarly, the ∑LREE/∑HREE values in different components of the leaf cell wall decreased in the order of HC2 > HC1 > pectin, with values of 1.77, 0.99, and 0.81, respectively (Figure 3). These results indicate that the binding behavior of REEs in different cell wall components of *P. americana* varies significantly and that the specific fractions of cell walls have certain selectivity for the adsorption of REEs.

### 2.3. Time-Dependent Adsorption Kinetics of REEs on the Cell Wall of P. americana

**Figure 4:**
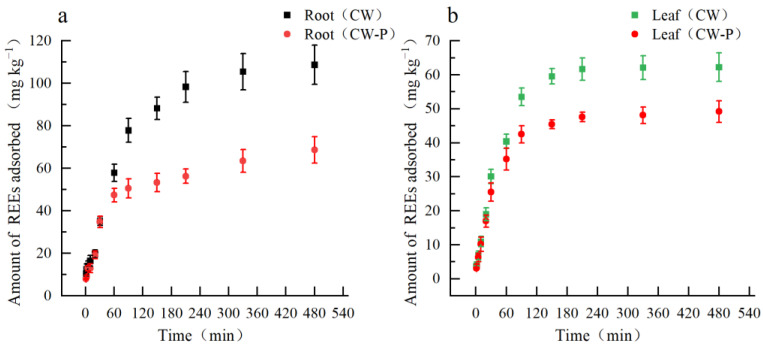
REEs adsorption kinetics of root (**a**) and leaf (**b**) cell walls with or without pectin in *Phytolacca americana*. “Root (CW)” and “Leaf (CW)” refer to the root and leaf cell walls. “Root (CW-P)” and “Leaf (CW-P)” refer to the root and leaf cell walls without pectin.

The adsorption of REEs by root and leaf cell walls can be characterized by three distinct stages, as shown in Figure 4. During the first stage (1–60 min), the adsorption rate of REEs by root cell walls rapidly increased, followed by a slower increase during the second stage (60–330 min), and reached equilibrium after 330 min, where the adsorption at equilibrium was 97% of the maximum adsorption (Figure 4a). Similarly, during the first stage (1–30 min), the adsorption rate of REEs by leaf cell walls rapidly increased, followed by a slower increase during the second stage (30–150 min), and reached equilibrium after 150 min, where the adsorption at equilibrium was 96% of the maximum adsorption (Figure 4b). Furthermore, the maximum adsorption capacity of REEs by root cell walls was significantly higher than that of leaves. Upon the removal of pectin, the adsorption capacity of REEs by root and leaf cell walls decreases significantly, with the maximum adsorption capacity decreasing by 39.8% and 23.6%, respectively.

### 2.4. Analysis of FTIR Spectroscopy for the Cell Wall of P. americana

**Figure 5:**
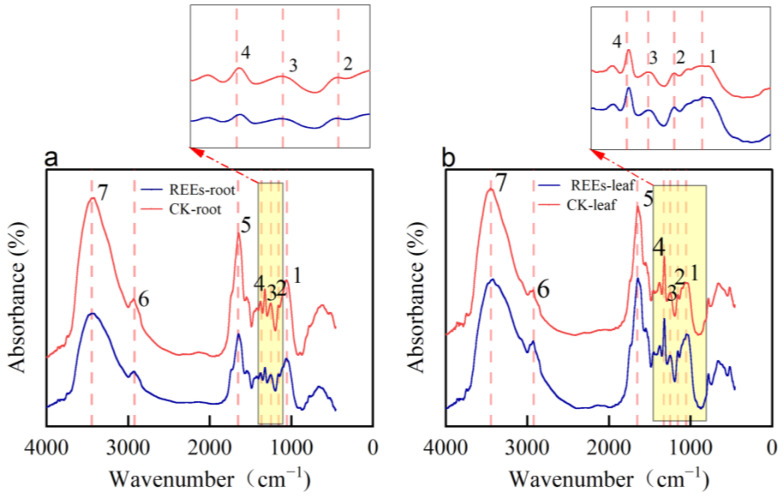
The Fourier transform infrared spectrometer (FTIR) spectra of root (**a**) and leaf (**b**) cell wall with and without REEs treatment. The color of the line represents the different treatments: the red line indicates treatments without REEs; the blue line indicates treatments with REEs. The numbers indicate different peak positions. Yellow boxes are partial enlargements.

The identification of functional groups in the cell wall that bind with REEs was achieved indirectly by observing the shift of FTIR peaks before and after REEs stress. In response to REEs stress, the FTIR characteristic peaks of both root and leaf cell walls were detected at 1053 cm^−1^ (1), 1155 cm^−1^ (2), 1249 cm^−1^ (3), 1327 cm^−1^ (4), 1652 cm^−1^ (5), 2922 cm^−1^ (6), and 3445 cm^−1^ (7) (Figure 5). Among these peaks, the peaks of amino acid-NH, sugar-OH (7), amide I band C-N (5), -C-O (4), carboxyl C-O (3), and pectin polysaccharide substance C-C or C-O (1) in the root cell wall showed a certain degree of shift towards the low-frequency direction. Notably, the -NH/-OH peak and C-C/C-O peak exhibited the most significant shift, with values of 29 cm^−1^ and 12 cm^−1^, respectively. On the other hand, the C-N peak (5), -C-O peak (4), and C-C/C-O peak (1) in the leaf cell wall exhibited a minor shift towards the low-frequency direction, with values of 2 cm^−1^, 10 cm^−1^, and 6 cm^−1^, respectively (Table 1).

### 2.5. Effect of REEs on the Total Sugar Content in Cell Wall Components of P. americana

**Figure 6:**
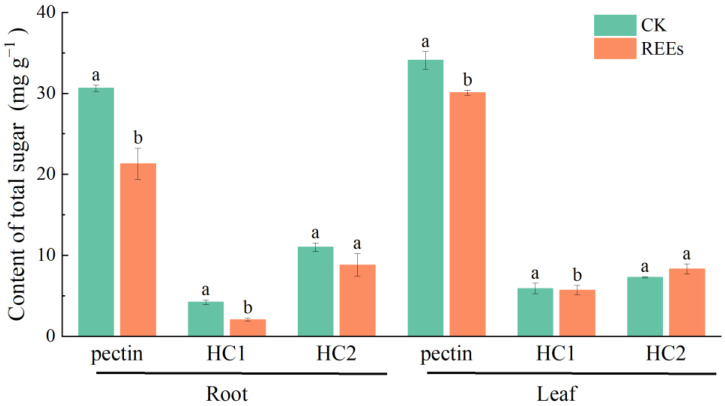
Total sugar content in different components (Pectin, Hemicellulose 1 (HC1), Hemicellulose 2 (HC2)) of root and leaf cell wall before and after REEs. The letter indicates the statistical difference between the control group (CK) and the REEs treated group (REEs) (ANOVA, *p* < 0.05).

The impact of REEs stress on the total sugar content of polysaccharide components in the cell wall of *P. americana* was investigated (Figure 6). The total sugar content in the pectin of root and leaf cell walls was substantially higher than that of HC1 and HC2. Under REEs stress, compared to the control, the total sugar content in the pectin, HC1, and HC2 fractions of the root cell wall was reduced by 30%, 51%, and 20%, respectively. In contrast, the total sugar content in the pectin and HC1 fractions of the leaf cell wall was reduced by 13% and 4%, respectively, under REEs stress.

### 2.6. Effect of REEs on the Galacturonic Acid Content in Cell Wall Components of P. americana

**Figure 7:**
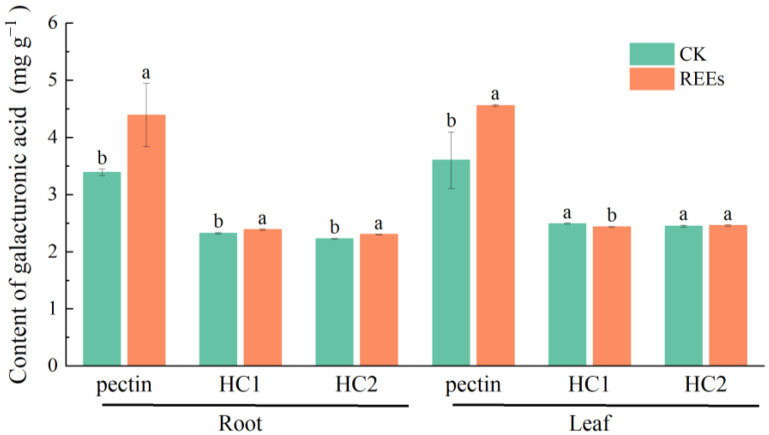
Total Galacturonic acid content in different components (Pectin, Hemicellulose 1 (HC1), Hemicellulose 2 (HC2)) of root and leaf cell wall before and after REEs. The letter indicates the statistical difference between the control group (CK) and the REEs treated group (REEs) (ANOVA, *p* < 0.05).

The exposure to REEs stress has been shown to induce alterations in the galacturonic acid content of polysaccharide components located in the cell wall of *P. americana* (Figure 7). Notably, the highest galacturonic acid content was observed in the pectin fraction of both the root and leaf cell walls. Following REEs treatment, the galacturonic acid content of the pectin, HC1, and HC2 fractions of both root and leaf cell walls exhibited an increase compared to the control. Particularly, the most notable increase in galacturonic acid content was observed in the pectin fraction of both root and leaf cell walls, where the increment was 68% and 27%, respectively. Nonetheless, no significant differences in the galacturonic acid content of HC1 and HC2 fractions were observed compared to the control.

## 3. Discussion

### 3.1. The Role of Cell Wall Polysaccharides of P. americana in REEs Adsorption

The cell wall is a highly complex structure comprising pectin, hemicellulose, proteins, lignin, and cellulose, possessing diverse functional groups, such as carboxyl, sulfhydryl, and hydroxyl groups, that could bind to metal ions [34,35,36]. A growing body of research has established that the cell wall serves as an important storage site for REEs in plants. For instance, studies have demonstrated that 81–88% of LREEs in *Dicropteris dichotoma* bind to the cell wall, while only 10% are associated with the cell membrane [37]. Leaf cell walls were reported to contain up to 56% of the total cell REEs [38], while REEs are predominantly stored in the cell wall of *Pediastrum simplex*, primarily in cellulose and pectin proteins [39]. Additionally, the subcellular distribution of Ce in rice roots revealed that most of the Ce is deposited in the cell wall [40]. The primary binding sites for metals in different plant cell walls vary. Studies have shown that pectin and hemicellulose are the principal binding sites for metals in cell walls. For instance, in soybean and pak choi, pectin accounts for 50% and 79%, respectively, of the CW-bound Cd content [41,42]. Similarly, 42–45% and 60–73% of the total Cd in root cell walls is deposited in the hemicellulose of the roots in *S. alfredii* and ramie [43,44]. Our research found that root and leaf pectin contained the highest REEs content, accounting for 41% and 48% of their total cell wall polysaccharide fractions, respectively. Following the removal of pectin, REEs adsorption in cell walls decreased significantly, with the maximum adsorption declining by 39.8% and 23.6%, respectively. These results suggested that pectin serves as the primary binding site for REEs in the cell walls of *P. americana*.

The capacity of the cell wall to bind metal ions is determined by the number of adsorption sites of its cations or the number of free carboxyl groups available [45,46]. Pectin, being a complex polysaccharide rich in a substantial quantity of functional groups such as carboxyl, hydroxyl, amino, and aldehyde, serves as an essential binding site for REEs adsorption [47,48]. After the removal of pectin, the functional groups on the cell walls were reduced, leading to a decrease in the binding sites available for REEs ions. Additionally, studies have indicated that the degree of methyl esterification of cell wall pectin is also a critical factor in its capacity to bind metal ions. The lower the degree of methylation, the more free carboxyl (-COOH) groups in pectin, therefore, enhancing the binding sites available for metal ions [49,50,51,52,53]. When pectin is incorporated into cell walls, it is typically highly methylated and subsequently demethylated by PME, resulting in the exposure of a large number of free carboxyl groups that serve as the primary binding sites for metal cations in cell walls [42]. In addition, exposure to heavy metals leads to an increase in PME activity, resulting in the observation of more low-methoxylated pectin in CWs as well as a lower degree of pectin methyl esterification, thereby increasing the number of metal ion binding sites in the cell wall [54]. The high concentration of rare earth elements (REEs) in pectin in this study may be due to the induction of REEs on low-methylated pectin in the cell wall. However, the mechanism, especially the molecular mechanism, of pectin demethylation in the CWs of *P. americana* ginseng roots is worth further investigation.

### 3.2. Fractionation of REEs in Different Fractions in the Cell Wall of P. americana

The binding of REEs in plant subcellular components varies among different substances, including cell walls, mitochondria, ribosomes, and chloroplasts. For example, in previous studies, approximately 56% of REEs were bound to the leaf cell wall of *Dicranopteris dichotoma*, while the remaining 44% were bound to other components within the cell, such as proteins or lipids [38]. The concentration of REEs in proteins was found to be much higher than that in the cell wall of *P. simplex* roots, with a REE-binding peptide exhibiting a higher affinity for LREEs [39]. Additionally, significant LREEs enrichment was observed in the leaf cell wall of *Dicropteris dichotoma* [37]. In this study, the enrichment of HREEs was observed in all fractions of the root cell wall (pectin, HC1, and HC2), whereas LREEs were enriched in all fractions of the leaf cell wall. This could be attributed to the root cell wall’s large number of carboxyl or hydroxyl groups, which can effectively bind to HREEs, while other LREEs pass through the cell wall and combine with other components inside the cell, such as proteins or lipids. The proportion of LREEs that entered the cell was found to be higher than those bound to the cell wall, while the distribution of LREEs and HREEs between leaf and root cells was the opposite. The distribution and compartmentalization of REEs at the subcellular level in plants collectively determine the extent of plant stress caused by REEs, which is one of the reasons why various REEs fractionation patterns occur among different organs in the cell walls of *P. americana*.

### 3.3. FTIR Analysis of Cell Wall in P. americana

Fourier transform infrared (FTIR) spectroscopy is a useful tool to analyze specific functional groups, such as hydroxyl, carboxyl, and methyl ester groups, in organic materials [55]. When plants are exposed to REEs stress, the functional groups in the cell wall bind with REEs, causing changes in peak intensity and peak position in the FTIR spectrum [56]. Previous studies have indicated that the adsorption and binding of metal ions in the cell wall depend on the number of cation adsorption sites and the type of functional groups present [57]. For example, the accumulation of Mn in the root cell walls of *P. americana* is linked to the presence of carboxyl, hydroxyl, and ester functional groups [58]. Additionally, the ability to bind with the Cd of hemicellulose’s root cell wall is largely influenced by the presence of hydroxyl, carboxyl, and carboxylate groups [22]. In this study, the results showed that the fundamental chemical structure of the cell wall remained largely unchanged before and after REEs adsorption (Figure 5). However, significant changes in peak position and intensity were observed after REEs adsorption, indicating the involvement of certain functional groups in the binding of REEs. For instance, after the adsorption of REEs in the root cell walls, the peak intensity of hydroxyl (-OH) at 3448 cm^−1^ weakened and shifted significantly toward a lower frequency, suggesting that REEs may bind to O ligands on hydroxyl (-OH) groups. The peak near 1652 cm^−1^, corresponding to the amide I band [59], indicated that proteins in the cell wall, which contain mainly carboxyl groups, were also involved in the binding of REEs. Furthermore, the weakened and shifted peak at 1063 cm^−1^ after REEs adsorption suggested that -COOH in pectin and cellulose might also serve as potential adsorption sites for REEs during the adsorption process in root cell walls. Similarly, significant changes in peak intensity and the position of functional groups were observed in the leaf cell walls after REEs adsorption. The involvement of -OH/-NH, C-O in cellulose and C-C/C-O in pectin in the adsorption and fixation of REEs was suggested by these changes. Overall, FTIR spectroscopic analysis revealed that -OH and -COOH were the primary functional groups involved in REEs adsorption in the root and leaf cell walls of *P. americana*.

### 3.4. The Changes of Polysaccharides to REEs in the Cell Wall of P. americana

Heavy metal stress can lead to growth inhibition in plant cells, causing changes in the content of polysaccharide components in the cell wall [20]. Several studies have reported elevated levels of polysaccharides in the cell walls of plants treated with heavy metals such as Cu and Cd [26,54]. Under Al stress, there was also a significant increase in the total sugars and galacturonic acid content in the pectin, cellulose, and hemicellulose of wheat cell walls [32]. In this study, the changes in polysaccharide content in the cell wall before and after REEs stress were investigated. The results indicated that the content of total sugar in the polysaccharide fractions of the cell wall was mostly decreased under REEs stress, while the content of galacturonic acid was increased (Figure 6 and Figure 7). The decrease in total sugar may be attributed to the toxic effect of REEs, leading to the degradation and decomposition of cell walls, which inhibited the activity of cell wall synthetase, thus reducing the rate of cell wall synthesis and total sugar content [60,61]. Under REE stress, plants may adjust the proportion and structure of cell wall components by reducing the total sugar content in the cell wall to adapt to stress environments [62,63], which is a REEs stress-responsive. Furthermore, galacturonic acid, a vital constituent of pectin, contains numerous functional groups that can effectively bind with heavy metal ions, immobilizing them in the cell wall [36]. Our study has demonstrated the important role of pectin in the accumulation of REEs in the cell wall. Therefore, the increase of galacturonic acid content is primarily aimed at increasing the binding sites of REEs ions on the cell wall, thus enhancing the adsorption and fixation of REEs by the cell wall [42]. In conclusion, our results showed that the decrease in total sugar content as well as the increase in galacturonic acid content in the cell wall is a complex manifestation of multiple factors, suggesting a role for cell wall polysaccharides in a novel mechanism of resistance to REEs.

## 4. Materials and Methods

### 4.1. Seed Collection and Plant Cultivation

Seeds of *P. americana* were collected from a high concentration of REEs mining in Dingnan County, Jiangxi province (24°59′16″ N,115°2′37″ E) [17]. To break the dormancy, seeds were submerged in H_2_SO_4_ (98%) for 30 s and then disinfected with H_2_O_2_ (15%) for 15 min. Sterilized seeds were then sown onto filter papers and watered with deionized water until germination. The previous methods were used to transplant the germinated seeds into 1 L containers filled with Hoagland nutritional solution [17]. The nutritional solution was continuously aerated, and the pH was adjusted to 5.5 ± 0.1 with HCl-KOH.

### 4.2. Plant Treatment and Cell Wall Extraction

After a four-week pre-cultivation period in the nutrient solution, five plants with consistent size were selected and cultivated for an additional six days in a nutrient solution containing 50 μM of REEs. At harvest, the plants were divided into roots and leaves, and their fresh weights were recorded. The divided plant parts were washed twice with deionized water and thoroughly dried. Cell walls (CWs) from the roots and leaves of *P. americana* were extracted using the procedure outlined by Zhu, et al. [45] with slight adjustments. Briefly, 5 g of fresh plant roots (or leaves) were frozen using liquid nitrogen and ground into a fine powder. The resulting powder was then homogenized in 75% ethanol and stored on ice for 20 min. Next, the samples were centrifuged at 9000× *g* for 10 min, and the collected pellets were washed successively with acetone, methanol/chloroform (1:1, *v*/*v*), and methanol at 4 °C. The pellets obtained from each washing step were considered as crude CW, freeze-dried, and preserved at 4 °C for future use.

### 4.3. Extraction and Analysis of Cell Wall Polysaccharides

The crude CW was separated into three fractions, namely pectin, hemicellulose 1 (HC1), and hemicellulose 2 (HC2), following the method described by Li, et al. [54]. The CW was first subjected to double extraction using 15 mL of 0.5% ammonium oxalate buffer (with 0.1% NaBH_4_) under boiling conditions for 1 h per extraction. The resulting supernatants were combined and identified as the pectin fraction (P), while the pellets were washed twice with deionized water and centrifuged. The resulting freeze-dried pellets were considered as the pectin-free cell wall fraction (CW-P). The CW-P pellets were then subjected to triple extraction using 15 mL of 4% NaOH (with 0.1% NaBH_4_) at 25 °C for a total of 24 h. The resulting supernatants were combined and assigned as the HC1 fraction. Similar extraction with 15 mL of 24% NaOH (with 0.1% NaBH_4_) was performed to obtain the HC2 fraction. Finally, the extracts were stored at 4 °C for further use.

### 4.4. Time-Dependent Adsorption Kinetics of REEs by Cell Walls

Four series of samples were used for the experiments: root cell wall (CW), root cell wall without pectin (CW-P), leaf cell wall (CW), and leaf cell wall without pectin (CW-P). Freeze-dried cell walls (CWs) weighing 0.05 g were loaded into a solid-phase extraction column (3 mL). A 10 μmol L^−1^ solution of REEs was injected into the column at a flow rate of 0.8 mL min^−1^, followed by filtration through a 0.22 μm membrane filter. Samples were collected at different time points (1, 2, 5, 10, 20, 50, 120, 240, and 480 min), and the adsorption experiments were repeated three times. The REEs concentrations in the filtrate were determined using Inductively Coupled Plasma-Mass Spectrometry (ICP-MS, NexION 350D, PerkinElmer, Waltham, MA, USA). The amounts of REEs adsorbed on root and leaf CWs were calculated using the following equation [64]:

$$q_{e}=\frac{(C_{0}-C_{e})\times v\times 10^{-3}}{m}$$

where $q_{e}$ is for the amount of REEs adsorption. $C_{0}$ and $C_{e}$ represent the initial concentration and the sampling point concentration of REEs, respectively. v represents the volume of the REEs collected. m represents the weight of freeze-dried CWs.

The REEs time-dependent adsorption kinetics experimental steps for the pectin-free cell wall fraction (CW-P) were conducted in a similar manner as described above.

### 4.5. Fourier Transform Infrared (FTIR) Spectra Measurements

Two milligrams of dried root and leaf cell walls, respectively, were weighed and mixed with 200 mg KBr (1:100, *w*: *w*). The mixture was then pressed into pellets using an agate mortar and measured for infrared spectra under identical conditions in the FTIR sample compartment. All infrared spectra were acquired with a resolution of 4 cm^−1^, covering the range of 400–4000 cm^−1^.

### 4.6. Determination of Cell Wall Polysaccharides

To determine the total sugar content of pectin, hemicellulose 1 (HC1), and hemicellulose 2 (HC2), expressed as glucose equivalents, the phenol sulfuric acid method [65] was used. The spectrophotometer (AA900T, PerkinElmer) was employed to measure the absorbance value at 490 nm. In addition, the content of galacturonic acid in pectin, HC1, and HC2, expressed as glucose equivalents, was determined using the m-hydroxydiphenyl method [45]. The spectrophotometer (AA900T, PerkinElmer) was employed to measure the absorbance value at 520 nm.

### 4.7. Statistical Analysis

All data were analyzed statistically using SPSS software (version 23.0, SPSS Inc., Chicago, IL, USA). A one-way ANOVA was performed to evaluate statistical differences among the groups, followed by Fisher’s LSD post hoc tests. The data were presented as arithmetic means accompanied by standard deviations. Values that shared the same letter were deemed not to have any statistically significant differences (*p* < 0.05 and *p* < 0.01). Graphical work was produced using Origin 2020.5. 

## 5. Conclusions

In summary, this study provides new insights into the mechanisms of REEs adsorption and detoxification in plants and contributes to a theoretical basis for the bioremediation of REEs pollution. Pectin was found to be the primary binding site for REEs in both root and leaf cell walls of *P. americana* and played a critical role in the absorption and detoxification of REEs in the cell walls. Hydroxyl (-OH) and carboxyl (-COOH) groups in the cell wall interacted mainly with REEs ions, which were the main binding site for REEs. Under REEs stress, the decrease in total sugar content as well as the increase in galacturonic acid content in the cell wall is a complex manifestation of multiple factors, suggesting a role for cell wall polysaccharides in a novel mechanism of resistance to REEs. These findings advanced our understanding of REEs stress-responsiveness of plant cell walls and provided potential solutions for the bioremediation of REEs pollution.

## Figures and Tables

**Table 1:** Semi-quantitative analysis of FTIR spectra of root and leaf cell walls before and after REEs adsorption.

Number	Functional Group	Root Cell Wall			Leaf Cell Wall		
		Before	After		Before	After	
		Wavenumber (cm^−1^)	Wavenumber (cm^−1^)	Offset (cm^−1^)	Wavenumber (cm^−1^)	Wavenumber (cm^−1^)	Offset (cm^−1^)
1	C-C/ C-O	1073	1061	−12	1049	1043	−6
2	C-C/ C-O	-	-	-	1155	1155	0
3	C-O-S/C-O /C-O-P	1252	1245	−7	1247	1247	0
4	C-O	1321	1319	−2	1321	1311	−10
5	C-N	1647	1645	−2	1647	1645	−2
6	-CH_3_ /=CH_2_/O-H	2929	2929	0	2927	2927	0
7	-OH/ -NH	3448	3419	−29	3421	3450	29

## Data Availability

No new data were created or analyzed in this study. Data sharing is not applicable to this article.
